# Long-Term Impact of a Culturally Tailored Patient Navigation Program on Disparities in Breast Cancer Screening in Refugee Women After the Program's End

**DOI:** 10.1089/heq.2018.0104

**Published:** 2019-05-14

**Authors:** Sebastian A. Rodriguez-Torres, Anne Marie McCarthy, Wei He, Jeffrey M. Ashburner, Sanja Percac-Lima

**Affiliations:** ^1^ Harvard School of Public Health, Harvard University, Boston, Massachusetts.; ^2^ Division of General Internal Medicine, Massachusetts General Hospital, Boston, Massachusetts.; ^3^ Department of Medicine, Harvard Medical School, Boston, Massachusetts.

**Keywords:** preventative health, minority health, breast health, mammography, screening, patient navigation

## Abstract

**Purpose:** To examine the long-term effects of a patient navigation (PN) program for mammography screening tailored to refugee women and to assess screening utilization among these women after PN ended.

**Methods:** We assessed the proportion of patients completing mammography screening during the prior 2 years during 2012–2016 for refugee women who had previously received PN compared with that of English-speaking women cared for at the same health center during the same period, both overall and stratifying by age. We used logistic regression to compare screening completion between refugees and English speakers, adjusting for age, race, insurance status, number of clinic visits, and clustering by primary care physician and to test trends in screening over time.

**Results:** In 2012, the year when the funding for PN ceased, among 126 refugee women eligible for breast cancer screening, mammography screening rates were significantly higher among refugees (90.5%, 95% confidence interval [CI]: 83.5–94.7%) than among English speakers (81.9%, 95% CI: 76.2–86.5%, *p*=0.006). By 2016, screening rates decreased among refugee women (76.5%, 95% CI: 61.6–86.9%, *p*=0.023) but were not statistically significantly different from those among English-speaking women (80.5%, 95% CI: 74.4–85.3%, *p*=0.460). Screening prevalence for refugee women remained above the pre-PN program screening levels, and considerably so in women <50 years.

**Conclusion:** The culturally and language-tailored PN program for refugee women appeared to have persistent effects, with refugee women maintaining similar levels of mammography screening to English-speaking patients 5 years after the PN program's end.

## Introduction

Although mammography screening is widely available in the United States,^1^ there are significant racial/ethnic disparities in breast cancer survival^2–5^ and mortality.^3,6,7^ Immigrants, nonnative English speakers, and low-income women are more often diagnosed with breast cancer at a late less treatable stage,^4,8–15^ due, in part, to lower utilization of mammography screening.^16–25^ Refugees, who have all of these characteristics, are a particularly disadvantaged population likely to face numerous barriers to care and screening and face unique challenges.^18,26–28^ Refugees tend to delay screening and are more likely to have never had a mammogram compared with nonrefugee immigrants.^26,29,30^ Disparities in refugee screening can be due to lack of knowledge of preventive health, lack of exposure to health professionals in their home countries, the impact of war on health systems, as well as fear of medical procedures and racial discrimination.^31–39^ Many refugees suffer from post-traumatic stress disorder resulting from the events surrounding their emigration, which can consequently make refugees particularly vulnerable to disparities in health.^16^

Patient navigation (PN) has been shown to reduce disparities in cancer screening and follow-up.^40–42^ Our group, as well as others, has demonstrated that PN screening programs in refugee communities can address and ultimately eliminate disparities in mammography screening rates in the short term.^27,43,44^

The 3-year program at Massachusetts General Hospital Chelsea HealthCare Center (MGH Chelsea) used culturally and linguistically tailored navigators to reach out to Bosnian, Somali, and Arabic refugee women and navigated them to obtain breast cancer screening. PN promoted mammography screening in refugee populations and consequently increased mammography rates to levels equivalent to those measured in nonimmigrant and native English-speaking populations.^43^ However, securing funding for patient navigators outside of a research setting can be a considerable challenge, and the MGH Chelsea navigation program ended after the research program concluded. It is unclear to what extent short-term navigation yields stable and sizable improvements in screening utilization in vulnerable populations.

The purpose of the study is to determine whether the PN program's reduction of screening disparities has persisted for an additional 5 years in the cohort of refugee participants from the previous study. Our study evaluates whether benefits of a 3-year PN program extend beyond the length of the actual program.

## Methods

Details of the PN program among refugee women have been published previously.^43^ In brief, the refugee PN program began in April 2009 at MGH Chelsea, when participants were introduced to the PN program with culturally and linguistically appropriate educational materials about breast cancer screening. Later, a navigator matched by culture and language background contacted the patient by phone or in person. Navigators worked to remove patients' individual psychological and logistical barriers to screening. Navigators educated women about breast cancer and screening, helped to schedule appointments, called participants about appointments, organized logistics, addressed insurance issues, and even accompanied them to radiology suite for testing. The PN program funding ceased on March 31, 2012.

Our study period extended 5 years from 2012, the year the PN program ended, through 2016. Women were defined as eligible for screening if they were 40 years of age or older, had not undergone mastectomy, and continuously had a primary care physician at MGH Chelsea. The primary study outcome was the proportion of patients who completed a mammogram during the prior 2 years. Screening proportions among refugees were compared with screening proportions among English-speaking patients cared for at MGH Chelsea in 2012. Patients were included in analyses for each year in which they remained primary care patients at MGH Chelsea. Data on completion of mammograms were obtained from electronic medical records and billing records. The Partners HealthCare Institutional Review Board approved this study.

We tabulated and compared patient characteristics between the groups at baseline. For each calendar year, we compared the proportion of patients completing mammography screening during the prior 2 years among refugee women with that among English-speaking patients. In addition, we examined the primary outcome stratified by patient age (40–49 and ≥50 years) since United States Preventative Services Task Force screening recommendations differ for these two age groups. We used logistic regression to compare the differences in the proportions completing screening with the generalized estimating equations procedure to account for clustering by primary care physician (PROC GENMOD, SAS version 9.2; SAS Institute, Cary, NC). To control for differences in patient characteristics among groups, patient age, race, insurance status, and the number of clinic visits over the prior 3 years were included in the models as covariates. In addition, we tested the trends in screening rates from 2012 through 2016 for refugees and English speakers using a difference in differences analysis, testing the interaction of time and group, accounting for clustering by primary care physician and adjusting for the mentioned covariates (PROC GLIMMIX). All statistical tests were two sided with an alpha of 0.05.

## Results

In the baseline year, 2012, there were 126 refugee women eligible for breast cancer screening. Since our study population of refugees consisted of those who remained from the original PN study, the number of eligible refugee women can only remain the same or diminish every year as participants discontinued their use of the clinic or had mastectomies. Ninety-eight refugee women were patients at the study clinics during all 5 years of the study period. Among 126 women at baseline, 29 (23%) were Arabic speaking, 75 (59.5%) were Serbo-Croatian speaking (Bosnian), and 22 (17.6%) were Somali speaking. Over the same period, there were 1,538 English-speaking women eligible for breast cancer screening in the participating clinic. The average age at baseline was 53.6 years among refugee women and 54.9 years for English speakers. At baseline, refugee women had higher proportions of Medicaid as their insurance and had different racial distributions than English-speaking women (Table 1).

**Table 1.** 2012 Baseline Characteristics of the Study Population

	**Refugee**	**English speakers**
*N*	126	1538
Age, mean (SD)	53.62 (9.2)	54.9 (9.1)
Clinic visits for 3 years, mean (SD)	11.25 (7.3)	9.31 (6.81)
Race/ethnicity, *N* (%)		
White	95 (75.4)	989 (64.3)
Asian	1 (0.8)	33 (2.1)
Black	25 (19.8)	177 (11.5)
Hispanic	0 (0.0)	324 (21.1)
Other/unknown	5 (4.0)	15 (1.0)
Insured, *N* (%)		
Commercial	55 (43.7)	853 (55.5)
Medicaid	53 (42.1)	248 (16.1)
Medicare	17 (13.5)	394 (25.6)
Self-pay/other	1 (0.8)	43 (2.8)
Mammogram in past 2 years, *N* (%)		
Yes	114 (90.5)	1239 (80.6)
No	12 (9.5)	299 (19.4)
Language, *N* (%)		
Arabic	29 (23.0)	0 (0.0)
Bosnian	75 (59.5)	0 (0.0)
Somalian	22 (17.5)	0 (0.0)
English	0 (0.0)	1538 (100.0)

SD, standard deviation.

**FIG. 1.**
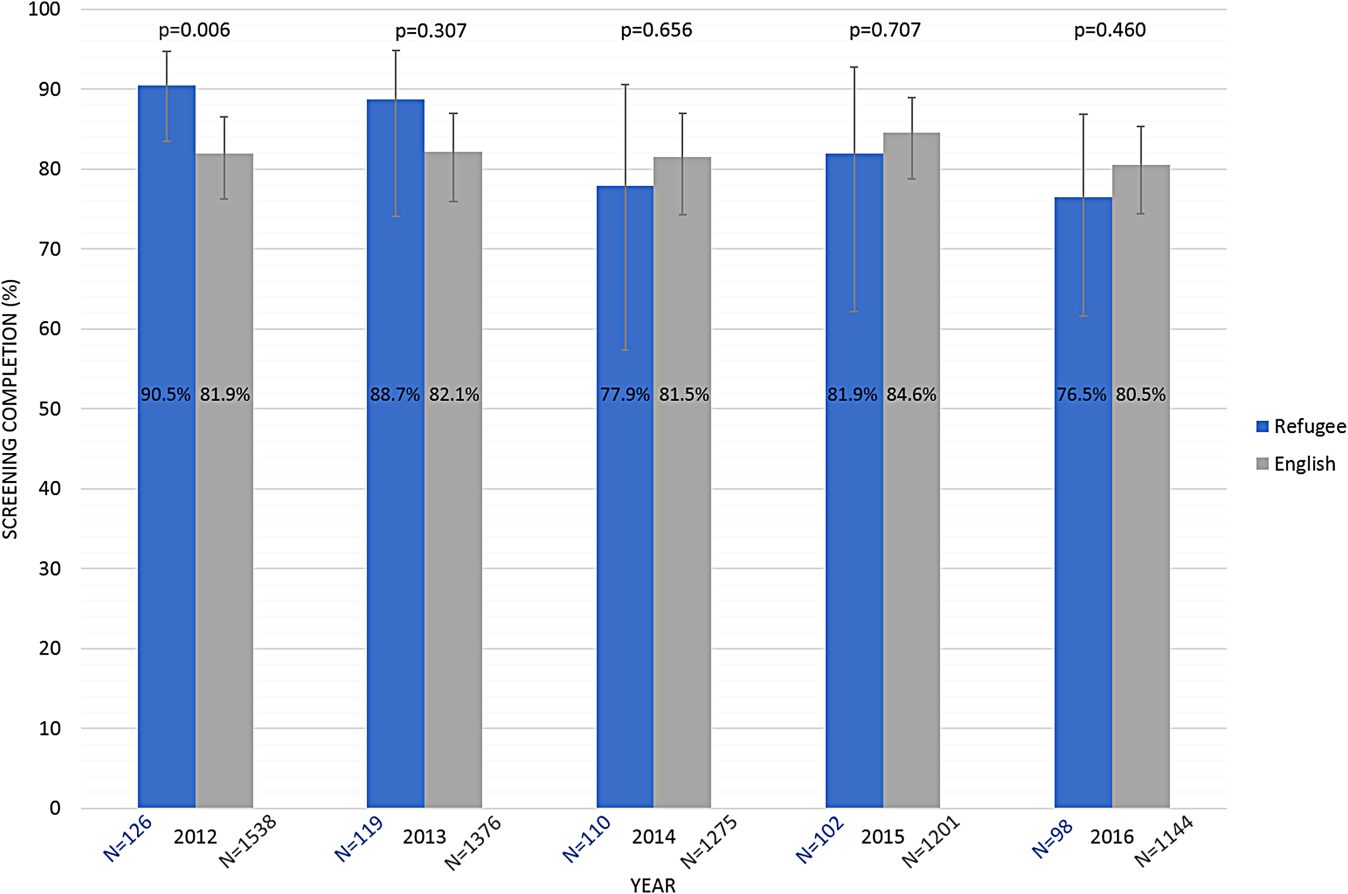
Mammography screening completion rate comparison. Mammography screening completion rates within the prior 2 years, along with sample size, *p*-values, and 95% confidence intervals for the English-speaking group compared with those of the referent group of refugees, calculated for each year for a 5-year period. Screening proportions are adjusted for patient age, race, insurance status, and number of clinic visits for the prior 3 years.

In 2012 when the PN program ended, mammography screening proportions were significantly higher among refugee women (90.5%, 95% confidence interval [CI]: 83.5–94.7%) than among English-speaking women (81.9%, 95% CI: 76.2–86.5%, *p*=0.006). After the PN program ended, the proportion of refugee women screened declined over time. There was a significant difference in the trends in screening rates over time for refugee women and English-speaking women (*p*=0.023). Although screening proportions among the English-speaking group did not change significantly over the 5-year period, screening rates among refugee women declined over time to 76.5% in 2016 (76.5%, 95% CI: 61.6–86.9. However, this rate was not statistically different from the screening proportion among English-speaking women (80.5%, 95% CI: 74.4–85.3%, *p*=0.460) (Fig. 1).

Stratifying results by patient age revealed that younger women (40–49 years) had a higher baseline prevalence of screening completion (96.4%) than the older (≥50 years) refugee women (86.8%), although both groups saw their screening completion prevalence decrease by a similar magnitude over the 5 years post-PN (11.9% for the ≥50 age group and 9.1% for the 40–49 age group). Among 40–49-year old women, screening prevalence was no different for refugees and nonrefugees, whereas for women aged 50 and older, screening prevalence was no different for refugees and English speakers. The greatest disparity in screening prevalence was seen among women of ages ≥50 years (Table 2).

**Table 2.** Adjusted Screening Proportions Stratified by Age

**40–49**					
	**Refugee**		**English**		
	**%**	**CI**	**%**	**CI**	***p***
2012	96.4	86.7–99.1	79.1	70.2–85.8	0.007
2013	92.7	84.9–96.6	79.7	70.3–86.5	0.001
2014	76.1	46.7–92.4	77.9	64.6–87.1	0.869
2015	83.2	52.4–95.8	80.6	66.0–89.7	0.811
2016	87.3	59.7–97.0	75.7	61.3–85.5	0.307

**50+**					
	**Refugee**		**English**		
	**%**	**CI**	**%**	**CI**	***p***
2012	86.8	73.5–93.9	84.1	76.0–89.7	0.506
2013	85.1	65.4–94.6	83.7	76.1–89.1	0.844
2014	79.0	60.3–90.4	83.2	75.9–88.6	0.555
2015	82.9	63.0–93.3	86.6	80.1–90.9	0.568
2016	74.9	57.8–86.6	82.5	76.0–87.5	0.178

CI, confidence interval.

## Discussion

We evaluated the persistence of reductions in disparities in breast cancer screening among refugee women for 5 years after a PN program's termination. We generally found decreases in screening completion for previously navigated refugee women in each year after the PN program ended, as is expected for periods after a PN program's end.^45^ However, in the fifth year after the PN program ended (2016), screening completion prevalence for refugee women was comparable with that of English-speaking primary care patients, and remained well above the prevalence of screening for refugee women before the PN program (76% vs. 64%).^43^ Screening rates for English-speaking women remained relatively stable over time, ranging from 77–82% in 2008–2011,^43^ and ranging from 81–85% for the years 2012–2016 after the refugee PN program's end. Our results suggest that even short-term PN interventions can have lasting effects.

Although multiple studies have documented effectiveness of PN for improving mammography screening utilization,^40,41,43,45–48^ to our knowledge no studies have documented outcomes after the program's end. It is well recognized that PN programs can be hard to maintain once research funding expires, and PN is typically not covered by health insurance, so PN programs need to be funded by research grants, philanthropic funds, or institutions.^49^ These insights into trends after PN program termination are important, particularly since one-time or limited duration interventions tend to be considerably less expensive than more involved continuous interventions.^50^

Previous studies have shown that even limited screening navigation interventions can have significant effects.^50,51^ Furthermore, the lasting effect after a program's end can play a great role in cost-effective calculations, since we show benefits of such a program can extend past the program's duration, and may be useful for assessing whether PN programs should be reimbursed as part of the routine care of vulnerable populations. PN may have persistent benefits due to increased patient knowledge about the importance of screening and familiarity with the mammography screening process. In addition, once patients receive a mammogram, radiology facilities at our site send letters to remind women when they are due for another mammogram, which may increase screening utilization.

The strengths of this study include our focus on refugee women, a particularly underserved and difficult to reach group. We were able to follow these women through electronic medical records for 5 years after the PN program ended to assess subsequent screening participation compared with nonrefugee women in the same health center. Limitations include that this study presents results using data from an academic medical center that may not be generalizable to other clinical settings. The screening rates we observed were high compared with national estimates. In 2015, 65.3% of women in the United States aged 40 years and over reported having a mammogram within the past 2 years.^52^ In our health system, primary care patients due for mammography screening received mailed reminders encouraging screening, which may partially account for high screening rates in our population.^53^ In addition, the decreasing number of refugees remaining in the sample each year decreased power to reject our null hypotheses as well as progressively increased the width of our prevalence CIs.

We cannot be certain that the PN program had truly persistent effects or whether comparable screening prevalence among refugee and nonrefugee women several years later reflects gradual acculturation into the health care system. A randomized study with a control group of non-navigated refugee women would be needed to determine whether screening participation among refugee women would increase to the levels of nonrefugee women over time without PN. Refugee women in our study had been in the United States for varying lengths of time. Bosnian refugees began arriving in the late 1990s, Somali immigrants began arriving around 2002, and Arabic-speaking refugees began arriving around 2005. In our prior study, PN was effective in increasing mammography screening among all three groups.^43^ Finally, the persistent effect of PN we observed for mammography screening may not extend to PN programs that focus on follow-up of abnormal screening tests or cancer treatment.

## Conclusion

The culturally and language-tailored PN program designed to reduce disparities in breast cancer screening among refugees appeared to have some lasting effect. Refugee women maintained higher prevalence of mammography completion compared with before PN and had screening rates similar to English-speaking women 5 years after the PN program's completion. The study revealed interesting trends in screening after the end of a PN program that could inform future program designs.

### Health equity implications

These findings suggest that culturally and language-tailored PN programs can have lasting positive effects on the women they serve. Refugee women are an underserved and vulnerable group within our society, who may be in particular need of the support provided through PN. The long-term benefits of PN observed in our study suggest that one time navigation might improve cancer outcomes over the long term.

## Abbreviations Used

CI: confidence interval
MGH Chelsea: Massachusetts General Hospital Chelsea HealthCare Center
PN: patient navigation
SD: standard deviation

## References

[1] Nelson HD, Tyne K, Naik A, et al. Screening for breast cancer: an update for the U.S. Preventive Services Task Force. Ann Intern Med. 2009;151:727–737, W237–W242. 19920273 10.1059/0003-4819-151-10-200911170-00009 PMC2972726

[2] Iqbal J, Ginsburg O, Rochon PA, et al. Differences in breast cancer stage at diagnosis and cancer-specific survival by race and ethnicity in the United States. JAMA. 2015;313:165–173 25585328 10.1001/jama.2014.17322

[3] Ellis L, Canchola AJ, Spiegel D, et al. Racial and ethnic disparities in cancer survival: the contribution of tumor, sociodemographic, institutional, and neighborhood characteristics. J Clin Oncol. 2018;36:25–33 29035642 10.1200/JCO.2017.74.2049 PMC5756323

[4] Keegan TH, Quach T, Shema S, et al. The influence of nativity and neighborhoods on breast cancer stage at diagnosis and survival among California Hispanic women. BMC Cancer. 2010;10:603 21050464 10.1186/1471-2407-10-603 PMC2988754

[5] Warner ET, Tamimi RM, Hughes ME, et al. Racial and ethnic differences in breast cancer survival: mediating effect of tumor characteristics and sociodemographic and treatment factors. J Clin Oncol. 2015;33:2254–2261 25964252 10.1200/JCO.2014.57.1349 PMC4486344

[6] Akinyemiju T, Moore JX, Ojesina AI, et al. Racial disparities in individual breast cancer outcomes by hormone-receptor subtype, area-level socio-economic status and healthcare resources. Breast Cancer Res Treat. 2016;157:575–586 27255533 10.1007/s10549-016-3840-x PMC4912843

[7] Whitman S, Orsi J, Hurlbert M The racial disparity in breast cancer mortality in the 25 largest cities in the United States. Cancer Epidemiol. 2012;36:e147–e151 22443886 10.1016/j.canep.2011.10.012

[8] Silva A, Molina Y, Hunt B, et al. Potential impact of the Affordable Care Act's preventive services provision on breast cancer stage: a preliminary assessment. Cancer Epidemiol. 2017;49:108–111 28601783 10.1016/j.canep.2017.05.015 PMC5544537

[9] Cho YI, Johnson TP, Barrett RE, et al. Neighborhood changes in concentrated immigration and late stage breast cancer diagnosis. J Immigr Minor Health. 2011;13:9–14 20232147 10.1007/s10903-010-9339-3

[10] Yasmeen S, Xing G, Morris C, et al. Comorbidities and mammography use interact to explain racial/ethnic disparities in breast cancer stage at diagnosis. Cancer. 2011;117:3252–3261 21246529 10.1002/cncr.25857

[11] Dosch AR, Koru-Sengul T, Miao F, et al. Racial and ethnic disparities in the diagnosis of breast cancer: changes in presenting stage in minority populations in Florida during 1981–2009. Breast Cancer Res Treat. 2014;148:379–387 25301087 10.1007/s10549-014-3158-5

[12] Oliveira K, Clark S, Dunn E, et al. Spanish as a primary language and its effect on breast cancer presentation. J Oncol Pract. 2011;7:165–167 21886497 10.1200/JOP.2010.000130 PMC3092656

[13] Kouri EM, He Y, Winer EP, et al. Influence of birthplace on breast cancer diagnosis and treatment for Hispanic women. Breast Cancer Res Treat. 2010;121:743–751 19949856 10.1007/s10549-009-0643-3

[14] MacKinnon JA, Duncan RC, Huang Y, et al. Detecting an association between socioeconomic status and late stage breast cancer using spatial analysis and area-based measures. Cancer Epidemiol Biomarkers Prev. 2007;16:756–762 17416767 10.1158/1055-9965.EPI-06-0392

[15] Lantz PM, Mujahid M, Schwartz K, et al. The influence of race, ethnicity, and individual socioeconomic factors on breast cancer stage at diagnosis. Am J Public Health. 2006;96:2173–2178 17077391 10.2105/AJPH.2005.072132 PMC1698157

[16] Kawar LN Barriers to breast cancer screening participation among Jordanian and Palestinian American women. Eur J Oncol Nurs. 2013;17:88–94 22459258 10.1016/j.ejon.2012.02.004

[17] Nonzee NJ, Ragas DM, Ha Luu T, et al. Delays in cancer care among low-income minorities despite access. J Women's Health. 2015;24:506–514 10.1089/jwh.2014.4998 PMC4490771 26070037

[18] Vahabi M, Lofters A, Kumar M, et al. Breast cancer screening disparities among immigrant women by world region of origin: a population-based study in Ontario, Canada. Cancer Med. 2016;5:1670–1686 27105926 10.1002/cam4.700 PMC4944895

[19] Goel MS, Wee CC, McCarthy EP, et al. Racial and ethnic disparities in cancer screening the importance of foreign birth as a barrier to care. J Gen Intern Med. 2003;18:1028–1035 14687262 10.1111/j.1525-1497.2003.20807.x PMC1494963

[20] Bhargava S, Moen K, Qureshi SA, et al. Mammographic screening attendance among immigrant and minority women: a systematic review and meta-analysis. Acta Radiol. 2018;59:1285–1291 29451023 10.1177/0284185118758132

[21] Yao N, Hillemeier MM Disparities in mammography rate among immigrant and native-born women in the U.S.: progress and challenges. J Immigr Minor Health. 2014;16:613–621 23430466 10.1007/s10903-013-9798-4 PMC3772964

[22] White A, Thompson TD, White MC, et al. Cancer screening test use—United States, 2015. MMWR Morb Mortal Wkly Rep. 2017;66:201–206 28253225 10.15585/mmwr.mm6608a1 PMC5657895

[23] Calo WA, Vernon SW, Lairson DR, et al. Area-level socioeconomic inequalities in the use of mammography screening: a multilevel analysis of the Health of Houston Survey. Womens Health Issues. 2016;26:201–207 26809487 10.1016/j.whi.2015.11.002 PMC4761271

[24] Kim J, Jang SN Socioeconomic disparities in breast cancer screening among US women: trends from 2000 to 2005. J Prev Med Public Health. 2008;41:186–194 18515996 10.3961/jpmph.2008.41.3.186

[25] Reyes-Ortiz CA, Markides KS Socioeconomic factors, immigration status, and cancer screening among Mexican American women aged 75 and older. Health Care Women Int. 2010;31:1068–1081 21058091 10.1080/07399332.2010.499183 PMC3032635

[26] Wu T-Y, Park Y Demographic predictors and cancer screening among Asian Americans in Michigan: role of refugee status. J Racial Ethn Health Disparities. 2017;4:770–777 28643271 10.1007/s40615-017-0397-2

[27] Gondek M, Shogan M, Saad-Harfouche FG, et al. engaging immigrant and refugee women in breast health education. J Cancer Educ. 2015;30:593–598 25385693 10.1007/s13187-014-0751-6 PMC4745125

[28] Murray KE, Mohamed AS, Ndunduyenge GG Health and prevention among East African women in the US. J Health Care Poor Underserved. 2013;24:233 23377731 10.1353/hpu.2013.0029 PMC3773084

[29] Barnes DM, Harrison CL Refugee women's reproductive health in early resettlement. J Obstet Gynecol Neonatal Nurs. 2004;33:723–728 10.1177/0884217504270668 15561660

[30] Morrison TB, Wieland ML, Cha SS, et al. Disparities in preventive health services among Somali immigrants and refugees. J Immigr Minor Health. 2012;14:968–974 22585311 10.1007/s10903-012-9632-4

[31] Saadi A, Bond BE, Percac-Lima S Bosnian, Iraqi, and Somali refugee women speak: a comparative qualitative study of refugee health beliefs on preventive health and breast cancer screening. Womens Health Issues. 2015;25:501–508 26219676 10.1016/j.whi.2015.06.005

[32] Kawar LN Jordanian and Palestinian immigrant women's knowledge, affect, cultural attitudes, health habits, and participation in breast cancer screening. Health Care Women Int. 2009;30:768–782 19657816 10.1080/07399330903066111

[33] Kobetz E, Menard J, Barton B, et al. Barriers to breast cancer screening among Haitian immigrant women in Little Haiti, Miami. J Immigr Minor Health. 2010;12:520–526 20091231 10.1007/s10903-010-9316-x

[34] Koh C, Nelson JM, Cook PF Evaluation of a patient navigation program. Clin J Oncol Nurs. 2011;15):41–48 10.1188/11.CJON.41-48 21278040

[35] Ellis BH, MacDonald HZ, Lincoln AK, et al. Mental health of Somali adolescent refugees: the role of trauma, stress, and perceived discrimination. J Consult Clin Psychol. 2008;76:184–193 18377116 10.1037/0022-006X.76.2.184

[36] Carroll J, Epstein R, Fiscella K, et al. Knowledge and beliefs about health promotion and preventive health care among somali women in the United States. Health Care Women Int. 2007;28:360–380 17454183 10.1080/07399330601179935

[37] Saadi A, Bond B, Percac-Lima S Perspectives on preventive health care and barriers to breast cancer screening among Iraqi women refugees. J Immigr Minor Health. 2012;14:633–639 21901446 10.1007/s10903-011-9520-3

[38] Harcourt N, Ghebre RG, Whembolua G-L, et al. Factors associated with breast and cervical cancer screening behavior among African immigrant women in Minnesota. J Immigr Minor Health. 2014;16:450–456 23334709 10.1007/s10903-012-9766-4 PMC3644538

[39] Dastjerdi M The case of Iranian immigrants in the greater Toronto area: a qualitative study. Int J Equity Health. 2012;11:9 22369146 10.1186/1475-9276-11-9 PMC3305531

[40] Phillips CE, Rothstein JD, Beaver K, et al. Patient navigation to increase mammography screening among inner city women. J Gen Intern Med. 2011;26:123–129 20931294 10.1007/s11606-010-1527-2 PMC3019333

[41] Marshall JK, Mbah OM, Ford JG, et al. Effect of patient navigation on breast cancer screening among African American medicare beneficiaries: a randomized controlled trial. J Gen Intern Med. 2016;31:68–76 26259762 10.1007/s11606-015-3484-2 PMC4700012

[42] Freund KM, Battaglia TA, Calhoun E, et al. Impact of patient navigation on timely cancer care: the Patient Navigation Research Program. J Natl Cancer Inst. 2014;106:dju115 24938303 10.1093/jnci/dju115 PMC4072900

[43] Percac-Lima S, Ashburner JM, Bond B, et al. Decreasing disparities in breast cancer screening in refugee women using culturally tailored patient navigation. J Gen Intern Med. 2013;28:1463–1468 23686510 10.1007/s11606-013-2491-4 PMC3797343

[44] Dunn SF, Lofters AK, Ginsburg OM, et al. Cervical and breast cancer screening after CARES: a community program for immigrant and marginalized women. Am J Prev Med. 2017;52:589–597 28094134 10.1016/j.amepre.2016.11.023

[45] Clark CR, Baril N, Kunicki M, et al. Addressing social determinants of health to improve access to early breast cancer detection: results of the Boston REACH 2010 Breast and Cervical Cancer Coalition Women's Health Demonstration Project. J Womens Health (Larchmt). 2009;18:677–690 19445616 10.1089/jwh.2008.0972

[46] Molina Y, Kim SJ, Berrios N, et al. Patient navigation improves subsequent breast cancer screening after a noncancerous result: evidence from the patient navigation in medically underserved areas study. J Womens Health (Larchmt). 2018;27:317–323 28933653 10.1089/jwh.2016.6120 PMC5865251

[47] Braun KL, Thomas WL Jr, Domingo JL, et al. Reducing cancer screening disparities in medicare beneficiaries through cancer patient navigation. J Am Geriatr Soc. 2015;63:365–370 25640884 10.1111/jgs.13192 PMC4850231

[48] Drake BF, Tannan S, Anwuri VV, et al. A community-based partnership to successfully implement and maintain a breast health navigation program. J Community Health. 2015;40:1216–1223 26077018 10.1007/s10900-015-0051-z PMC4626535

[49] Freund KM Implementation of evidence-based patient navigation programs. Acta Oncol. 2017;56:123–127 28033027 10.1080/0284186X.2016.1266078

[50] Ladabaum U, Mannalithara A, Jandorf L, et al. Cost-effectiveness of patient navigation to increase adherence with screening colonoscopy among minority individuals. Cancer. 2015;121:1088–1097 25492455 10.1002/cncr.29162 PMC4558196

[51] Glassgow AE, Molina Y, Kim S, et al. A comparison of different intensities of patient navigation after abnormal mammography. Health Promot Pract. 2018 [Epub ahead of print]; DOI: 10.1177/1524839918782168 PMC6274628 29907079

[52] National Center for Health and Statistics. Health, United States, 2017: With Special Feature on Mortality. Hyattsville, Maryland: National Center for Health Statistics, 2018 30702833

[53] Atlas SJ, Ashburner JM, Chang Y, et al. Population-based breast cancer screening in a primary care network. Am J Manag Care. 2012;18:821–829 23286611 PMC3766952

